# Supplementary material for: Clinical PD-1/PD-L1 Blockades in Combination Therapies for Lymphomas
Source: Cancers (Basel). 2023 Nov 14;15(22):5399. doi: 10.3390/cancers15225399 (PMC10670854; doi:10.3390/cancers15225399)
Supplement: Supplementary file 1 [file cancers-15-05399-s001.zip › cancers-2613190-supplementary.pdf]

Supplementary Table S1. The current status of clinical studies on combination therapies involving PD-1/PD-L1 blockade in lymphomas.

Diseases	NCT number	PD-1/PD-L1 blockade	Combined agents	Phases	Study Status
**Classical Hodgkin lymphoma**					
cHL	NCT03233347	Nivolumab	BV-AVD	2	Active, not recruiting
cHL	NCT03004833	Nivolumab	AVD	2	Active, not recruiting
cHL	NCT05772624	Nivolumab	AVD	2	Recruiting
cHL	NCT04091490	Nivolumab	DHAP	2	Recruiting
cHL	NCT03033914	Nivolumab	A(B)VD	1/2	Recruiting
cHL	NCT05723055	Nivolumab	Axatilimab	2	Recruiting
cHL	NCT04134325	Nivolumab	CD30 CAR-T	1	Recruiting
cHL	NCT05675410	Nivolumab	ABVD or BV	3	Recruiting
cHL	NCT02408861	Nivolumab	Ipilimumab	1	Recruiting
cHL	NCT03681561	Nivolumab	Ruxolitinib	1	Active, not recruiting
cHL	NCT05039073	Nivolumab	BV-AVD	2	Recruiting
cHL	NCT02758717	Nivolumab	BV	2	Active, not recruiting
cHL	NCT03739619	Nivolumab	Gemcitabine+Bendamustine	1/2	Active, not recruiting
cHL	NCT03907488	Nivolumab	BV-AVD	3	Active, not recruiting
cHL	NCT04938232	Nivolumab	Ipilimumab	2	Recruiting
cHL	NCT05660993	Nivolumab	Bendamustine+Gemcitabine+ Vinorelbine	2	Recruiting
cHL	NCT03016871	Nivolumab	ICE	2	Active, not recruiting
cHL	NCT04561206	Nivolumab	BV	2	Recruiting
cHL	NCT03057795	Nivolumab	BV	2	Active, not recruiting
cHL	NCT03138499	Nivolumab	BV	3	Terminated
cHL	NCT03712202	Nivolumab	BV	2	Active, not recruiting

cHL	NCT02940301	Nivolumab	Ibrutinib	2	Active, not recruiting
cHL	NCT02927769	Nivolumab	BV	2	Active, not recruiting
cHL	NCT05352828	Nivolumab	CD30 CAR-T	1	Active, not recruiting
cHL	NCT04981899	Nivolumab	ICE	1/2	Recruiting
cHL	NCT05508867	Pembrolizumab	Favezelimab	3	Recruiting
cHL	NCT04788043	Pembrolizumab	Magrolimab	2	Recruiting
cHL	NCT05180097	Pembrolizumab	BV	2	Recruiting
cHL	NCT04134325	Pembrolizumab	CD30 CAR-T	1	Recruiting
cHL	NCT02684292	Pembrolizumab	BV	3	Active, not recruiting
cHL	NCT05355051	Pembrolizumab	Azacitidine	2	Recruiting
cHL	NCT03471351	Pembrolizumab	Tenalisib	1	Terminated
cHL	NCT03776864	Pembrolizumab	Umbralisib	2	Terminated
cHL	NCT05404945	Pembrolizumab	BV or AVD	2	Recruiting
cHL	NCT03226249	Pembrolizumab	AVD	2	Active, not recruiting
cHL	NCT05922904	Pembrolizumab	BV-AVD	2	Not yet recruiting
cHL	NCT04510636	Pembrolizumab	Bendamustine	2	Recruiting
cHL	NCT05008224	Pembrolizumab	ABV or BEACOPP	2	Active, not recruiting
cHL	NCT03331341	Pembrolizumab	AVD	2	Active, not recruiting
cHL	NCT03077828	Pembrolizumab	ICE	2	Active, not recruiting
cHL	NCT04044222	Sintilimab	ICE	3	Recruiting
cHL	NCT05502250	Tislelizumab	Gemcitabine+Cisplatin	2	Recruiting
cHL	NCT04843267	Tislelizumab	AVD	2	Recruiting
cHL	NCT05137886	Tislelizumab	Decitabine	2	Recruiting
cHL	NCT04067037	Camrelizumab	AVD	2	Recruiting
cHL	NCT04233294	Camrelizumab	Chidamide+Decitabine	2	Recruiting
cHL	NCT04510610	Camrelizumab	Chidamide+Decitabine	2/3	Recruiting
cHL	NCT04514081	Camrelizumab	Chidamide+Decitabine	2	Recruiting
cHL	NCT03617666	Avelumab	ABVD	2	Active, not

					recruiting
cHL	NCT05300282	Atezolizumab	Bendamustine+Gemcitabine+ Vinorelbine	1/2	Not yet recruiting
**B-cell lymphoma**					
B-cell lymphoma	NCT03995147	Pembrolizumab	R-CHOP	2	Recruiting
B-cell lymphoma	NCT03892525	Atezolizumab	Selicrelumab	1	Terminated
B-cell lymphoma	NCT03533283	Atezolizumab	Glofitamab	1/2	Recruiting
B-cell lymphoma	NCT03321643	Atezolizumab	R-GemOx	1	Active, not recruiting
DLBCL/PCNSL	NCT03558750	Nivolumab	Rituximab+Lenalidomide	1	Terminated
DLBCL/PMBCL	NCT03484819	Nivolumab	Copanlisib Hydrochloride	2	Active, not recruiting
DLBCL	NCT05385263	Nivolumab	CD19 CAR-T	2	Recruiting
DLBCL/FL	NCT03401853	Pembrolizumab	Rituximab+Obinutuzumab	2	Active, not recruiting
DLBCL/FL	NCT02446457	Pembrolizumab	Rituximab+Lenalidomide	2	Recruiting
DLBCL	NCT05221645	Pembrolizumab	R-ICE	2	Recruiting
DLBCL	NCT03309878	Pembrolizumab	Mogamulizumab	1/2	Active, not recruiting
DLBCL	NCT05280626	Sintilimab	R-CHOP	2	Not yet recruiting
DLBCL	NCT04659434	Sintilimab	R-GemOx	2	Not yet recruiting
DLBCL	NCT04058470	Toripalimab	R-CHOP	1/2	Recruiting
DLBCL	NCT05659628	Tislelizumab	CD19 CAR-T	1	Recruiting
DLBCL	NCT05093140	Camrelizumab	R-CHOP	2	Not yet recruiting
DLBCL/Mantle	NCT03440567	Avelumab	Utomilumab+R-ICE	1	Active, not recruiting
DLBCL	NCT02951156	Avelumab	Utomilumab+Rituximab± azacitidine or bendamustine	3	Terminated
DLBCL	NCT03244176	Avelumab	R-CHOP	1	Active, not recruiting
DLBCL/MCL/FL	NCT03685344	Durvalumab	Loncastuximab Tesirine	1	Terminated
DLBCL	NCT03422523	Atezolizumab	R-GemOx	2	Terminated
PMBCL	NCT04759586	Nivolumab	R-DA-EPOCH	3	Recruiting
PMBCL	NCT04745949	Nivolumab	BV-R-CHP	2	Recruiting

PMBCL	NCT05934448	Pembrolizumab	CAR-T	2	Not yet recruiting
PMBCL	NCT04705129	Tislelizumab	Zanubrutinib	2	Recruiting
PCNSL	NCT04609046	Nivolumab	Lenalidomide	1	Recruiting
PCNSL	NCT03770416	Nivolumab	Ibrutinib	2	Active, not recruiting
PCNSL	NCT04421560	Pembrolizumab	Ibrutinib+Rituximab	1/2	Recruiting
PCNSL	NCT04961515	Sintilimab	Orelabrutinib	1/2	Recruiting
PCNSL	NCT04899427	Sintilimab	Orelabrutinib	2	Recruiting
PCNSL	NCT04899427	Tislelizumab	Orelabrutinib	2	Recruiting
PCNSL	NCT04688151	Durvalumab	Rituximab+Acalabrutinib	1	Not yet recruiting
PCNSL	NCT04462328	Durvalumab	Acalabrutinib	1	Recruiting
Aggressive B-cell lymphoma	NCT03038672	Nivolumab	Varlilumab	2	Active, not recruiting
Aggressive B-cell lymphoma	NCT03749018	Nivolumab	DA-EPOCH-R	2	Active, not recruiting
High-grade B-cell Lymphoma	NCT03620578	Nivolumab	DA-EPOCH-R	2	Active, not recruiting
FL	NCT03245021	Nivolumab	Rituximab	1	Active, not recruiting
FL	NCT04962126	Atezolizumab	Obinutuzumab	2	Active, not recruiting
Indolent Lymphoma	NCT04431635	Nivolumab	Copanlisib	1	Active, not recruiting
CLL	NCT03204188	Pembrolizumab	Ibrutinib+Fludarabine	2	Active, not recruiting
CLL/MCL	NCT03153202	Pembrolizumab	Ibrutinib	1/2	Recruiting
CLL/Richter	NCT04781855	Nivolumab	Ipilimumab+Ibrutinib	1	Recruiting
CLL/Richter	NCT05388006	Durvalumab	Acalabrutinib+Venetoclax	2	Recruiting
CLL/Richter	NCT02846623	Atezolizumab	Obinutuzumab+Venetoclax	2	Recruiting
Richter syndrome or transformed FL	NCT03892044	Nivolumab	Duvelisib	1	Active, not recruiting
Richter syndrome or transformed indolent lymphoma	NCT03884998	Nivolumab	Copanlisib	1	Recruiting

**T-cell lymphoma**					
PTCL	NCT03927105	Nivolumab	Cabiralizumab	2	Active, not recruiting
PTCL	NCT03586999	Nivolumab	DA-EPOCH	1/2	Active, not recruiting
PTCL	NCT05313243	Pembrolizumab	BV	2	Recruiting
PTCL	NCT03278782	Pembrolizumab	Romidepsin	1/2	Active, not recruiting
PTCL	NCT04795869	Pembrolizumab	BV	2	Active, not recruiting
PTCL	NCT03598998	Pembrolizumab	Pralatrexate	1/2	Active, not recruiting
PTCL	NCT04052659	Sintilimab	Chidamide+Azacitidine	2	Not yet recruiting
PTCL	NCT04512534	Sintilimab	Chidamide	2	Recruiting
PTCL/CTCL	NCT03240211	Pembrolizumab	Decitabine+Pralatrexate	1	Recruiting
PTCL/CTCL	NCT03011814	Durvalumab	Lenalidomide	1/2	Recruiting
CTCL	NCT05956041	Pembrolizumab	Mogamulizumab	2	Not yet recruiting
CTCL	NCT04296786	Sintilimab	Chidamide	2	Recruiting
Mycosis Fungoides/Sezary Syndrome	NCT04960618	Pembrolizumab	Gemcitabine	2	Recruiting
Mycosis Fungoides/Sezary Syndrome	NCT03063632	Pembrolizumab	Interferon-gamma	2	Active, not recruiting
NK/T-cell lymphoma	NCT02535247	Pembrolizumab	Copanlisib	1/2	Terminated
NK/T-cell lymphoma	NCT04365036	Toripalimab	Pegaspargase-GemOx	3	Recruiting
NK/T-cell lymphoma	NCT04899414	Tislelizumab	Azacytidine+Pegaspargase	2	Not yet recruiting
NK/T-cell lymphoma	NCT05464433	Tislelizumab	Mitoxantrone Hydrochloride Liposome	1/2	Recruiting
NK/T-cell lymphoma	NCT05316246	Tislelizumab	BV	2	Not yet recruiting
NK/T-cell	NCT05058755	Tislelizumab	Azacytidine+Lenalidomide	NA	Recruiting

lymphoma					
NK/T-cell lymphoma	NCT05254899	Tislelizumab	Pegaspargase-GemOx	2	Recruiting
NK/T-cell lymphoma	NCT04366128	Camrelizumab	Pegaspargase+Apatinib+RT	NA	Recruiting
NK/T-cell lymphoma	NCT04994210	Sintilimab	Chidamide	2	Recruiting
NK/T-cell lymphoma	NCT04676789	Sintilimab	Pegaspargase	2	Not yet recruiting
NK/T-cell lymphoma	NCT04127227	Sintilimab	Pegaspargase+GemOx	2	Recruiting
**Lymphoid neoplasm**					
Lymphoma	NCT03502733	Nivolumab	Ipilimumab	1	Active, not recruiting
Lymphoma	NCT01703949	Nivolumab	BV	2	Recruiting
Lymphoma	NCT03015896	Nivolumab	Lenalidomide	1/2	Active, not recruiting
Lymphoma	NCT03366272	Nivolumab	R-GemOx	2/3	Active, not recruiting
Lymphoma	NCT05255601	Nivolumab	Relatlimab	1/2	Recruiting
Lymphoma	NCT03150329	Pembrolizumab	Vorinostat	1	Active, not recruiting
Lymphoma	NCT03179930	Pembrolizumab	Entinostat	2	Recruiting
Lymphoma	NCT03598608	Pembrolizumab	Favezelimab	1/2	Recruiting
Lymphoma	NCT02875067	Pembrolizumab	Lenalidomide	1/2	Terminated
Lymphoma	NCT02362035	Pembrolizumab	Acalabrutinib	1/2	Active, not recruiting
Lymphoma	NCT05320081	Camrelizumab	CD30 CAR-T	2	Recruiting
Lymphoma	NCT03636503	Avelumab	Utomilumab+Rituximab	1	Active, not recruiting
Lymphoma	NCT03161223	Durvalumab	Pralatrexate+Romidepsin+ Oral 5-Azacitidine	1/2	Recruiting
Lymphoma	NCT02500407	Atezolizumab	Mosunetuzumab	1/2	Active, not recruiting

Abbreviations: cHL, classical Hodgkin lymphoma; DLBCL, diffuse large B-cell lymphoma; PMBCL, primary mediastinal B-cell lymphoma; PCNSL, primary central nervous system lymphoma, FL, follicular lymphoma; CLL, chronic lymphocytic leukemia; MCL, mantle cell lymphoma; PTCL, peripheral T-cell lymphoma; CTCL,

cutaneous T cell lymphoma; BV, brentuximab vedotin; AVD, doxorubicin, vinblastine, and dacarbazine; ICE, ifosfamide, carboplatin, and etoposide; DHAP, dexamethasone, high dose cytarabine, and cisplatin; CAR-T, chimeric antigen receptor T-cell; R, rituximab; CHOP, cyclophosphamide, doxorubicin, vincristine, and prednisolone; GemOx, gemcitabine and oxaliplatin; DA-EPOCH, dose adjusted-cyclophosphamide, doxorubicin, etoposide, vincristine, and prednisolone; RT, radiotherapy; NA, not available
